# An Optimal Time for Treatment—Predicting Circadian Time by Machine Learning and Mathematical Modelling

**DOI:** 10.3390/cancers12113103

**Published:** 2020-10-23

**Authors:** Janina Hesse, Deeksha Malhan, Müge Yalҫin, Ouda Aboumanify, Alireza Basti, Angela Relógio

**Affiliations:** 1 Institute for Theoretical Biology (ITB), Charité—Universitätsmedizin Berlin, Corporate Member of Freie Universität Berlin, Humboldt—Universität zu Berlin and Berlin Institute of Health, 10117 Berlin, Germany; janina.hesse@charite.de (J.H.); deeksha.malhan@charite.de (D.M.); muege.yalcin@charite.de (M.Y.); ouda.aboumanify@charite.de (O.A.); alireza.basti@charite.de (A.B.); 2 Molecular Cancer Research Center (MKFZ), Medical Department of Hematology, Oncology and Tumor Immunology, Charité—Universitätsmedizin Berlin, Corporate Member of Freie Universität Berlin Humboldt—Universität zu Berlin and Berlin Institute of Health, 10117 Berlin, Germany; 3 Department of Human Medicine, Institute for Systems Medicine and Bioinformatics, MSH Medical School Hamburg—University of Applied Sciences and Medical University, 20457 Hamburg, Germany

**Keywords:** chronotherapy in cancer, core-clock ODE models, circadian time prediction, machine learning, harmonic regression, computational methods for rhythmicity analysis, circadian network

## Abstract

**Simple Summary:**

Personalized cancer treatments show decreased side-effects and improved treatment success. One aspect of individualized treatment is the timing of medicine intake, which may be optimized based on the biological diurnal rhythm of the patient. The personal biological time can be assessed by a variety of tools not yet commonly included in diagnostics. We review these tools with a focus on their applicability in a clinical context. Using biological samples from the patient, most tools predict individual time using machine learning methodologies, often supported by rhythmicity analysis and mathematical core-clock models. We compare different approaches and discuss possible promising future directions.

**Abstract:**

Tailoring medical interventions to a particular patient and pathology has been termed personalized medicine. The outcome of cancer treatments is improved when the intervention is timed in accordance with the patient’s internal time. Yet, one challenge of personalized medicine is how to consider the biological time of the patient. Prerequisite for this so-called chronotherapy is an accurate characterization of the internal circadian time of the patient. As an alternative to time-consuming measurements in a sleep-laboratory, recent studies in chronobiology predict circadian time by applying machine learning approaches and mathematical modelling to easier accessible observables such as gene expression. Embedding these results into the mathematical dynamics between clock and cancer in mammals, we review the precision of predictions and the potential usage with respect to cancer treatment and discuss whether the patient’s internal time and circadian observables, may provide an additional indication for individualized treatment timing. Besides the health improvement, timing treatment may imply financial advantages, by ameliorating side effects of treatments, thus reducing costs. Summarizing the advances of recent years, this review brings together the current clinical standard for measuring biological time, the general assessment of circadian rhythmicity, the usage of rhythmic variables to predict biological time and models of circadian rhythmicity.

## 1. Introduction

**Figure 1:**
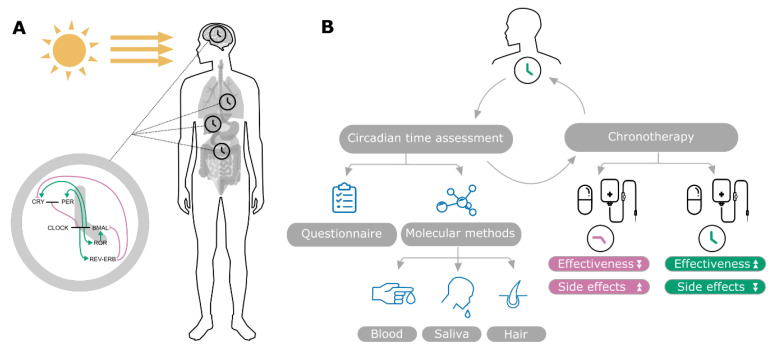
Circadian clock main components and the interplay between circadian time assessment and chronotherapy. (**A**) The internal clock is composed of central and multiple peripheral clocks, sharing a common molecular network of transcription/translation feedback loops. (**B**) Multiple methods are used to assess internal circadian rhythms including chronotype evaluation questionnaires and various biological samples that can be collected to assess hormonal concentration and gene expression profiles. Time-of-day treatment adjustment in alignment with the internal circadian clock of the body has been reported to increase effectiveness and decrease toxicity of treatment.

Cancer treatment typically aims to eradicate cancer cells, for example by inducing DNA damage and impairing DNA repair mechanisms and triggering cell death pathways while keeping side-effects at bay. Recent research shows that the efficacy of medication is influenced by the timing of the administration, known as chronotherapy [1]. The administration time can be best coordinated to the daily rhythm that modulates not only our sleep and wake phases but also physiological characteristics such as body temperature and heart rate or, more relevant to cancer treatment, immune response and xenobiotic detoxification pathways (Figure 1) [2,3,4]. Multiple clinical studies have successfully demonstrated the potential of chronotherapy as an effective treatment optimization tool, which could increase effectiveness, lower drug toxicity and increase survival. Supporting evidence is provided by randomized clinical trials that compared chronotherapy to conventional treatment especially in the case of metastatic colorectal cancer [5,6,7]. 

**Figure 2:**
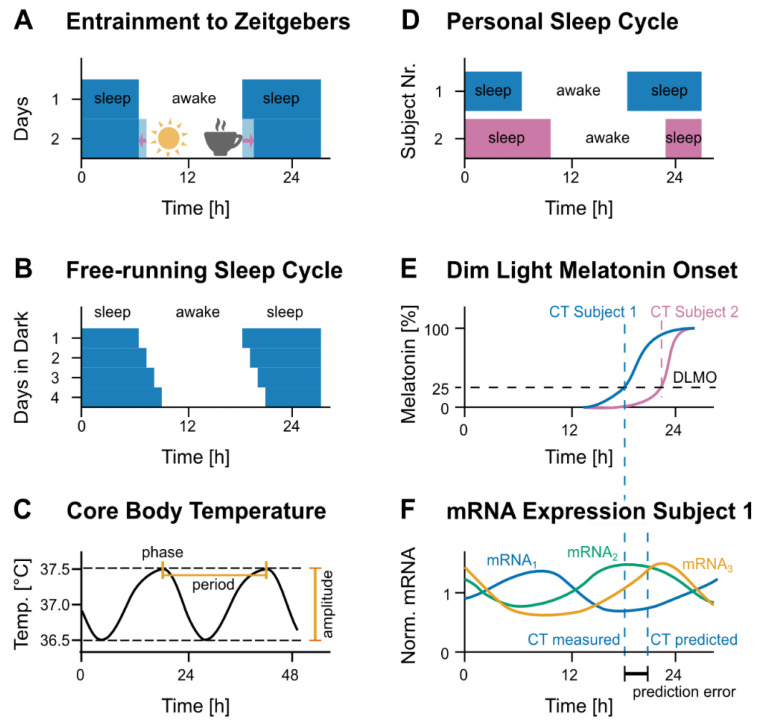
Circadian oscillations in humans. (**A**) The circadian rhythm is evident from the sleep/wake cycle of humans. Subjected to a normal day-night rhythm, humans are awake (white) and sleep (blue) with a period adapted to 24 h. Normally, the first light acts as a strong zeitgeber that entrains the subject to its rhythm. Other zeitgebers include temperature, food or the cup of coffee in the evening, which likely delays sleep onset. (**B**) Under constant light conditions, humans retain a rhythm of wake (white) and sleep (blue) phases with a period of around 24 h. In this example, the period is slightly longer than 24 h, thus shifting wake and sleep from day to day. (**C**) The core body temperature shows circadian oscillations. The phase (also named acrophase) is defined as the first peak of the oscillation, the time between two peaks is the period and the amplitude is the difference between peak and trough. (**D**) Indicated are the awake (white) and sleep (colored) phases for two subjects. Subject 1 (blue) has an earlier phase than subject 2 (pink). (**E**) To measure the dim-light melatonin onset (DLMO), subjects are kept under dim-light conditions during the evening, with hourly sampling for subsequent melatonin quantification. The circadian phase is defined as the time when the concentration of melatonin reaches an absolute (e.g., 2 pg/mL) or relative (e.g., 25%) threshold [15]. (**F**) The analysis of circadian oscillations of a set of genes for a given subject (here visualized with profile sketches for three imaginary transcripts, mRNA_1_, mRNA_2_, mRNA_3_) allows to predict the circadian time of that subject. The difference between predicted and actual circadian time constitutes the prediction error.

Daily rhythms or circadian rhythms (from the Latin *circa dies, about a day*), are generated by an endogenous timing mechanism and differ between subjects. This timing mechanism is sensitive to external signals such as light, temperature, food intake or a cup of coffee (so-called zeitgebers), thus aligning (entraining) the organism to the geophysical time (Figure 2A). The geophysical time is related to the zeitgeber time, which is for humans often defined as sunrise and in general based on external signals such as day and night. The circadian time, on the other hand, is the subjective time of the organism. Each morning, the first light of the day acts as a strong zeitgeber and resets the circadian time to the geophysical time, a process named entrainment (Figure 2A). Entrained to the day-night rhythm, the time interval between two peaks of the rhythm (period of the oscillation) amounts to circa 24 h [8]. Circadian oscillations are endogenously generated and persist with a period of around 24 h even in the absence of external cues. Under constant environmental conditions (controlled light exposure), the circadian free-running period (Figure 2B,C) in humans may differ by up to one hour [9]. The different internal periods correlate with preferential wakeup times, subjects with a short period tend to wake up about 3 h earlier than subjects with a long period (Figure 2D) [10,11], seemingly in accordance with differences in the rhythmicity of core-clock genes of up to three hours [12]. The subjective morning can thus differ by a couple of hours between two subjects. The difference in circadian time can get even larger throughout the day, as a shorter period effectively speeds up the passing of time. 

Circadian oscillations are commonly characterized by three parameters, the above-mentioned period, the amplitude of the oscillation (the difference between the maximum expression and the minimum expression values) and the phase, which is, for a circadian oscillation, the time of the first maximum of expression (peak of expression) within 24 h (Figure 2C). To assess the phase, the maximum of the data points is a crude estimate, especially for data with a low temporal resolution, for example, one data point every three hours. Instead, the phase and other parameters of interest, such as amplitude and period, are often extrapolated using a cosine function fitted to the data instead of the data itself (Section 3.1).

In practice, there is no agreed-upon definition of circadian time. In principle, circadian time should denote the absolute phase of the central pacemaker, which is however not directly measurable in humans. In lieu, the phase of several physiological oscillations, as reviewed below, are used to define circadian time, most common are definitions based on a subject’s melatonin profile (Figure 2E). Subtracting phases of different subjects, we obtain their difference in circadian time [13], sometimes referred to as phase difference, which is a better defined quantity. More informative than a single number representing circadian time might be the circadian profile, that is, measurements of physiological parameters over 24 h. It is worth noticing that an alternative definition of circadian time identifies the start of an organism’s activity as circadian time zero and passing of time is measured in circadian hours defined as 1/24 of the organism’s period under dark-dark conditions. This definition is however hardly useful in humans for whom the activity schedule is shaped rather by society than by biology. If the circadian time is used, instead of geophysical time, treatment can be optimally personalized to the circadian rhythm of the patient. Indeed, knowledge of the circadian time or eventually of the full circadian profile of a subject, might also be useful to align all sorts of daily activities, from food intake to sports and sleep, to one’s internal rhythm and thus maintaining a good health, also in extreme environments (e.g., astronauts in space missions [14]).

In mammals, the circadian timing system is hierarchically regulated with two main levels of control, the central pacemaker clock and the peripheral clocks, located in most peripheral tissues including heart, liver, kidney and skin, controlled by a complex network of interacting genes and proteins [16]. The central pacemaker is located in the suprachiasmatic nuclei (SCN) within the anterior part of the hypothalamus. The SCN can be entrained directly via external cues (e.g., light) and generate cellular autonomous oscillations that propagate towards other tissues and organs (Figure 1A). Ablation of the SCN led to the loss of rhythm in hormonal secretion and physiological functions [17]. The SCN send synchronizing information to other cells in the body through physiologic cues (e.g., temperature), hormonal activity and the autonomous nervous system [18]. Peripheral clocks are also influenced by external signals such as food and activity, which have been investigated in depth in mammals [19].

Both the SCN clock and the peripheral clocks rely on the same molecular network, formed by interconnected transcription and translation feedback loops (TTFLs). The mechanistic insights on the TTFLs and the discovery of the molecular mechanisms controlling circadian rhythms led, in 2017, to the Nobel Prize in Physiology and Medicine awarded to Jeffrey C. Hall, Michael Rosbash and Michael W. Young [20,21,22]. In mammals, the core-clock is responsible for inducing circa 24 h oscillations mainly through a dynamic interplay between two feedback loops bound via the CLOCK/BMAL1 heterodimer complex [23]. CLOCK (circadian locomotor output cycles kaput) and BMAL1 (Brain and Muscle ARNT-Like1) activate the transcription of multiple target genes that play a key role in circadian regulation. Among those are elements of the negative feedback loop, PER1, PER2, PER3 (Period 1, 2 and 3) and CRY1, CRY2 (Cryptochrome 1 and 2), which inhibit CLOCK/BMAL1 function [24]. A second loop, which is activated by the CLOCK/BMAL1 complex regulates the expression of *BMAL1* via the nuclear receptors RORA, RORB, RORC (RAR related orphan receptors A, B and C) and REV-ERBα, REV-ERBβ (REV-ERB nuclear orphan receptors). The interplay between these two transcription/translation feedback loops orchestrates the circadian oscillatory mechanism in cells (Figure 1A). This intricate time keeping system plays a significant role in regulating various molecular and cellular functions from RNA processing to immune pathways, it controls several steps in metabolic pathways and governs the expression patterns of numerous cell cycle proteins [25,26,27,28,29,30,31,32]. Subsequently, disruption in circadian processes may not only favor cancer progression but also result in various other pathologies [33,34,35,36]. 

The influence of the circadian clock on health and disease has been extensively reviewed in recent work [37,38,39,40,41], here we review some of the methods that can be used to determine a personalized circadian time, with a focus on the mammalian system to facilitate clinical applicability. We start with a brief overview on the current developments of chronotherapy (Section 2) and then review the computational strategies for assessing circadian rhythms (Section 3). We describe how circadian rhythms can be computationally evaluated, how machine-learning can then be used to extract a personal circadian time and how mathematical models of the mammalian core-clock network can contribute to improve the estimate of circadian time. Finally, we discuss the translational potential and other aspects related to chronotherapy (Section 4). 

## 2. Clinical Overview

The circadian clock plays an instrumental role in human health and disease, as described in the Introduction. Due to its function as regulator of physiology and behavior in synchrony with external environmental cues, the circadian machinery is responsible for generating and maintaining proper organ functionality, via regulating gene and protein activity. In fact, studies conducted in animal models including mice [42] and the primate *Papio anubis* (baboon, [43]) reported that more than 80% of protein-coding genes show circadian rhythms in expression, in at least one tissue and several of these genes are involved in processes related to the so-called hallmarks of cancer [44]. These include genes governing key biological functions such as energy metabolism (e.g., *NR1D1*, *HKDC1*, *PCK1* and *GLUTs*), DNA repair (e.g., *XPA* and *TP53*), cell cycle (e.g., *MYC*, *WEE1* and *INK4A*), cell motility (e.g., *NR1D1* and *SNAI1)*, as well as protein and macromolecules integrity (e.g., *KRT1*, *ITM2B*) [25,28,42,43,45,46,47,48]. The circadian clock thus plays a central role in maintaining tissue homeostasis, which, if disrupted, could lead to disease onset or progression. Therefore, it is relevant to consider the bidirectional interplay between the internal clock of the patient and the applied therapy regimen accurately in order to enhance patient outcome and treat diseases more effectively [49].

### 2.1. Current Methods to Assess Circadian Time

It is of paramount importance to develop robust methods to monitor and accurately characterize the circadian time in humans. Multiple approaches can be used to tackle this aspect, each with its particular advantages and limitations. The term “chronotype” is used to denominate the relative phase of entrainment of the individual to the environment, that is, the temporal difference between the internal timing (e.g., morning vs. evening types) and external zeitgeber (e.g., light-dark cycles). The chronotype attempts to classify a subject as a “morning” or “evening” person [50]. Around 30% of individuals are classified as morning type, 20% as evening type while the remaining 50% are considered to have a chronotype in between the two extremes [51]. One of the simplest ways to estimate the chronotype is via a structured questionnaire. Multiple questionnaire-based methods have been developed for chronotype assessment including the Morningness-Eveningness Questionnaire (MEQ) [50], the Composite Scale of Morningness (CSM) [52] and the Munich ChronoType Questionnaire (MCTQ) [53], which vary in the categorization of the participants in their morning-evening preference type and in the number of questions included. Additionally, shorter forms of the questionnaires have been developed, based on the same principles but incorporating less questions [54]. More information regarding the number of questions and the type of results acquired from the questionnaires are provided in Table 1.

While questionnaires serve as a quick and simple method to assess chronotypes and determine Morningness-Eveningness preferences, they lack the robust objective perspective of an experimental method and do not provide insights into the molecular basis leading to the different chronotypes. To overcome these limitations multiple experimental techniques using biological samples have been developed and provide a more comprehensive and objective perspective [14]. The current gold standard method to determine the exact internal time in humans is referred to as DLMO (dim-light melatonin onset) [55]. In order to measure DLMO, blood, saliva or urine samples are collected every 0.5–1 h under dim light conditions, as light suppresses melatonin secretion and the concentration of the pineal hormone melatonin is measured [13]. DLMO (Figure 2C) is proven to be very accurate in assessing phase delays or advances of internal rhythms of an individual in relation to the external environment, which has an impact on health and performance [13,56]. Another hormone-based method to experimentally assess the circadian timing is measuring cortisol concentration, which also shows robust circadian variations [57]. Hormone-based measurements bare two main limitations: the inconvenience of the need for high-sampling frequency and the lack of information at the gene expression level. Thus, developing other approaches that could accurately determine the internal clock, more conveniently and using fewer time points is needed. Gene expression data accompanied with recent computational methods (discussed in detail in Section 3) present a valuable asset that could help solving this problem by gaining molecular insights into the circadian time. Moreover, gene expression-based methods provide direct information at the gene level, which can be further used to identify circadian drug targets and time-relevant aspects in drug metabolism related to drug delivery and efficacy. Measuring the expression of core-clock and/or clock-controlled genes involved in drug processing can be used to monitor the pharmacokinetic and pharmacodynamic interactions, which are also impacted by the oscillatory machinery. For instance, antiangiogenic agents, interferon and irinotecan hydrochloride are examples of drugs with varying efficiency and toxicity associated with the circadian rhythm of the patient where a deeper analysis at the gene expression level would be advantageous for treatment planning [58]. However, it remains unclear how measuring rhythmic gene expression from peripheral tissues contributes to the assessment of pharmacokinetic and pharmacodynamic interactions (see Discussion). Several biological materials have been used for the extraction of gene expression information. These are mainly (semi-) invasive methods, to determine peripheral clock phenotypes in humans via gene expression profiling [59]. Blood is commonly used (via invasive sampling methods) to assess the internal clock of individuals based on gene expression profiles [60,61,62,63]. Hair follicle cells and oral mucosa (semi-invasive sampling) have also been used to evaluate the peripheral circadian clock in humans based on the genetic expression profiles of clock genes [64,65,66].

With current advancements in technology and in particular in wearable devices, more attention is being taken toward using wearables to assess circadian and ultradian rhythms in humans (reviewed in [67]). A major advantage of using wearables is that they enable real-time and high-resolution monitoring of one’s circadian rhythms by tracking physiological indications (e.g., heart-rate, rest-activity, sleep, glucose, skin temperature and exposure to external cues such as light) [68,69,70,71]. The data obtained from one or multiple wearables can serve as high-dimensional input data to computational models or machine learning approaches in order to personalize chronotherapy for patients [72]. For cancer patients, for example, wearables can be used to assess the impact of chronomodulated drug delivery on the daily life and physiological parameters of patients in real time [73]. This can involve multidimensional tele-monitoring of circadian rest-activity rhythms (CircAct), sleep, patient-reported outcome measures and body weight changes (BWCs) [73].

### 2.2. Chronotherapy and Its Importance for Cancer Treatment

The recent advancements in the understanding of the molecular mechanisms underlying the circadian effects and the accumulating evidence on the fundamental role of the biological clock in health and disease, have driven researchers to further investigate the advantages of circadian regulation in a clinical setting [49]. One of the most promising applications of circadian biology is its potential in the optimization of drug administration regimen in alignment with the circadian rhythm of the patient, referred to as “chronotherapy” [74,75]. This is highlighted by the fact that around 50% of the top 100 best-selling drugs in the United States (among which are numerous drugs used for cancer treatment) and notably all the top 7 best-selling drugs, target proteins that are the products of circadian expressed genes (Table 2) [42]. All target genes listed in Table 2 show circadian oscillations according to the circaDB database (http://circadb.hogeneschlab.org/) in mouse tissues and 8 genes show circadian oscillations in human tissues, which may vary from tissue to tissue and patient to patient and thus need to be tested individually. 

A broad range of treatments for various medical conditions has been investigated in recent years including: allergy [76,77], arthritis [78,79], asthma [80,81], hyperlipidemia [82,83], hypertension [84,85] and cancer (Table 3). In particular, tyrosine kinase inhibitors such as Erlotinib, Sunitinib and Lapatinib targeting EGFR/Ras/Raf/MAPK pathway have shown chronopharmacological effects in mouse and rabbit models [86,87,88,89,90]. Also, Roscovitine (aka. Seliciclib, CDK inhibitor), Everolimus (mTOR inhibitor) and Irinotecan (Top1 inhibitor) all belong to anticancer drugs, which show time-of-day dependent effects in mouse models [91,92,93]. The concept of chronotherapy and its interplay with circadian time assessment including its different methods [94] is visualized in Figure 1. The raising potential of chronotherapy has motivated an increasing collection of clinical studies that have been conducted to examine the effect of time-of-day adjusted treatment [6,75,95,96,97,98]. Hence, the usage of time-of-day adapted therapies personalized with the internal clock of the patient might be superior to standard treatment, however the results are sometimes varying in terms of the desired effect of chronotherapy in comparison to standard treatment in particular concerning gender-related efficacy of chronotherapy [99,100]. More studies and a more precise stratification of patients are needed before chronotherapy can be used in the clinical practice.

## 3. Computational Methods 

As chronotherapy capitalizes on circadian rhythmicity, an adaptation of treatment to personalized circadian time instead of to external time should further strengthen its advantages (Figure 1). To facilitate the estimation of circadian time of a patient, several groups have proposed computational methods, which use as input time-course data from subjects to predict the circadian time. In the following, we will discuss how biological data is initially assessed for oscillations, which prediction algorithms have been used and how models can help to estimate the circadian time.

Data driven computational predictions are increasingly used in medical care. Among the used computational methods are machine-learning algorithms [121]. These derive an artificial relation between subject data and circadian time, which, when used on new data, can predict circadian time in a subject-specific way, for example, relating gene expression with circadian time as inferred from the melatonin profile [60,63]. For the prediction of circadian time, a machine-learning algorithm is trained on a set of inputs, for example, the patient’s gene expression data, with the circadian time as desired output. This set with known input and output is also called training set [121]. As research on human data often copes with small numbers of subjects, a successful algorithm should be able to generalize well despite the small training sets used. This is typically tested on a second set of data with known circadian time, the test set. The prediction of the algorithm based on the patient data is compared to the actually measured circadian time, allowing thereby to gauge the quality of the personalized prediction (Figure 2F) [60,122].

A personalized prediction of circadian time must be based on some form of patient data. To ensure a sufficient amount of information, two different types of data can be used, low-dimensional data with a high temporal resolution or high-dimensional data with a lower temporal resolution. High temporal resolution can be gained by wearable technical devices that continuously measure physiological data [123,124,125,126,127,128]. The high temporal resolution seems to balance the low number of features measured, for example, for temperature and light exposure data, in contrast to RNA sequencing data with thousands of transcripts measured. While two physiological parameters are sufficient for predicting circadian time (as defined by the melatonin profile) with a precision comparable to using more parameters, using a single parameter reduces prediction precision significantly [128].

Though these approaches result in relatively precise prediction of the circadian time [127,128], the need of special devices has so far limited their applicability in a clinical context. The availability of high-resolution data appropriate for predictions of circadian time will dramatically increase now that patients can acquire their own devices that track physiological parameters, such as smart watches.

An alternative approach is to use more complex data but with a low temporal resolution—often just a single-time point measurement. Gene expression data is sufficiently complex and can be extracted from blood or saliva samples (see Section 2, [64,65,66]). However, expression data from non-relevant genes might confuse the machine learning approach. Additional data that is non-informative on the circadian time enhances the amount of data required for training the prediction algorithm, as the algorithm needs to separate useful from useless data in addition to the prediction step itself. To optimize the data for machine learning, high-dimensional data is frequently reduced to a subset of allegedly informative data, often only data showing circadian rhythmicity is used. For physiological data such as skin temperature or activity, circadian variation is well-established. For gene expression data, there are several tools that distinguish between oscillating and non-oscillating genes, reviewed in Section 3.1. The selection and pre-processing of patient data, such as the detection of rhythmically expressed genes, is an important step in the prediction of circadian time, as it constrains the information available for the prediction. Once an appropriate data set for prediction has been selected, several well-established prediction mechanisms are available. Those used in previous studies will be reviewed in Section 3.2. The prediction mechanisms can be separated into model-based and model-free algorithms. Model-free algorithms ground the prediction solely on the data of the training set. On the other hand, model-based algorithms are equipped with additional biological information on the process, considering the underlying molecular regulatory network. We will review the potential benefit of models for the prediction of circadian time in Section 3.3.

### 3.1. Computational Methods for Detecting Rhythmic Patterns 

The circadian transcriptome is defined as the set of expressed genes, which show a rhythmic behavior in their mRNA expression levels with a periodicity of ~24 h. Early studies using microarrays revealed the existence of rhythmic gene expression profiles in various organisms from fruit flies to humans [129,130,131,132,133]. With the development of next generation sequencing (NGS) platforms, RNA-seq became one of the most commonly used high-throughput platforms for the generation of time-course expression data [29,30]. Other NGS platforms widely used in the field include chromatin immune precipitation DNA-sequencing (CHIP-seq) [134,135] for studying the transcriptional and chromatin regulation of clock-controlled rhythms and regulation of cycling mRNAs, which revealed links to biological processes (e.g., metabolism) [136]. The relevance of the experimental design for circadian experiments, as well as the parameters to be considered (e.g., sampling frequency, number of complete circadian cycles, number of replicates) for the measurements have been extensively discussed [137,138,139,140]. In particular, rhythm detection is facilitated by an appropriately chosen measurement protocol. For circadian studies, it is recommended that the experimental data is collected for more than a day to capture the rhythmic patterns (ideally over 2 consecutive days), with 2 to 3 h sampling interval. If the collection of data points over 48 h is not possible, the usage of a shorter time series might be compensated by the inclusion of biological replicates in the experimental design. 

However, the impact of the number of data points collected, in the oscillatory properties and in rhythmicity detection has not yet been fully explored. A previous study by Akashi and colleagues investigated the use of different number of data points for the assessment of circadian rhythmicity in humans, which were collected over one daily cycle (24 h) [141]. Hair follicles were used in the study as the biological source for the characterization of clock gene expression profiles. Samples were collected at different time intervals (3, 6, 9 and 15 h). The authors suggested that the prediction of circadian parameters was accurate using three-data points when samples were collected with 3 h and 6 h intervals. The results showed an accurate prediction of phases also using a single time point for nine core-clock genes. Hence, the authors recommended to design the sampling interval as short as possible (3 h) in order to conclude an accurate phase prediction that can be applicable to clinical use.

**Figure 3:**
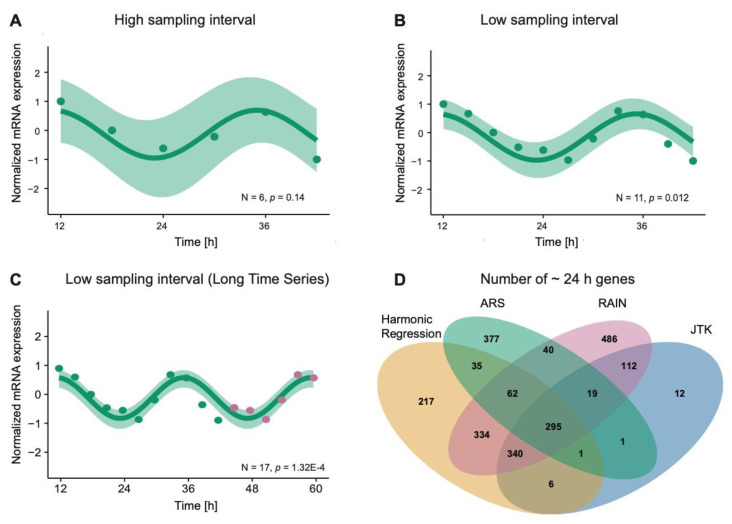
The impact of experimental design and computational algorithm of choice for the assessment of circadian rhythmic expression. Fitted oscillation curves (green lines) together with the experimental values (full circles) were plotted using harmonic regression for *BMAL1* (in SW480 cells, E-MTAB-7779). (**A**) Normalized time-series expression for *BMAL1* with a low temporal resolution (N = 6; 6 h sampling interval). (**B**) Normalized time-series expression for *BMAL1* as measured experimentally in the time series used (N = 11; 3 h sampling interval). (**C**) Normalized time-series expression for *BMAL1* duplicating the data points (pink full circles) between 21–36 h (N = 17; 3 h sampling interval). The green area marks the confidence area of the harmonic regression fitted to the data for 24 h rhythmic expression. (**D**) Venn diagram for the visualization of the intersection of 24 h rhythmic expression detected by RAIN, JTK, Harmonic regression and ARS.

In this section, we aimed to extract rhythmic profiles in different experimental set ups and to analyze how the rhythmicity detection is affected based on the sampling interval and the number of data points. The sampling interval and the number of data points included in the experimental design are directly correlated to the statistical significance of the results. The increase in the number of data points results in lower *p*-values. To illustrate the relationship between the frequency of data collection and the robustness in rhythmicity detection, we used a previously published RNA-seq data set from the human colorectal cancer cell line SW480 (E-MTAB-7779, [29]). SW480 cells were synchronized by medium change and samples were collected from 12 to 42 h after synchronization with a sampling interval of 3 h [29]. We selected the core-clock gene *BMAL1* as an example for our analysis and used the harmonic regression [142] method to generate a fitting curve for the visualization of the rhythmic expression of *BMAL1* (Figure 3A–C). In this particular example, the harmonic regression produces a statistically significant fit (*p* = 0.012) in the 30 h time-course data collected with 3 h sampling interval (N_Timepoints_ = 11) (Figure 3B). To visualize the impact of sample size and sampling interval in the analysis, we plotted the oscillation of *BMAL1* by excluding data points (from the original data set), which resulted in a sampling interval of 6 h (N_Timepoints_ = 6) (Figure 3A). Lastly, to simulate a longer time-course data set, we duplicated the time points, which resulted in a virtual data set sampled over two -consecutive days (Figure 3C). As exemplified by these results, for the study of circadian rhythmicity, ideally the data should be sampled for longer than one day and with a short sampling frequency (3 h) (Figure 3C). For larger sampling intervals (6 h, Figure 3A) *BMAL1* was not detected as significantly oscillating (*p* = 0.14). Additionally, technical and biological replicates can increase the robustness of the results and the accurate assessment of statistical analysis [138].

Next, we analyzed the differential output between the different methods used for detecting rhythmic gene expression. The choice of the particular computational algorithm for detection of cycling genes and transcripts is a crucial aspect in the analysis of the circadian transcriptome. There are multiple algorithms available in the literature for the analysis of time-series data, which recognize different sets of genes as oscillating, see for example, Figure 3D. These methods vary mostly in terms of the input waveforms that they optimally analyze: symmetric waveforms (harmonic regression [142], ARSER [143], JTK_CYCLE and Lomb Scargle [144]) and asymmetric waveforms (RAIN [145] and empirical JTK_CYCLE [146]). Another important distinction can be made based on the type of output produced by the different methods: period and harmonics (Lomb Scargle periodogram [144] and spectral analysis with ARSER [143]); or other circadian properties like mesor, amplitude and phases (harmonic regression [142], JTK_CYCLE [147], BIO_CYCLE [148] and ARSER [143]). In the following, we described in detail each of these methods.

Gene expression values are provided as input for all the algorithms in order to study changes occurring at the transcriptome level over time. These inputs are then processed based on either frequency [149,150] or time-domain approaches [151,152]. For time-domain approaches, the time series is explored for the presence of a trend and a forecasting model is fitted by treating the events in the data as a function of time. The frequency-based approaches on the other hand aim at the deconvolution of periodic functions present in a time series by using wave-like periodic profiles, which can be modelled by cosine/sine functions. One of the most well-known procedures in the field for the detection of circadian rhythmicity is using harmonic regression, which is based on trigonometric functions. Another well-known method, RAIN [145] detects also non-parametric rhythmic patterns and can deal with missing values, as well as outliers. RAIN detects symmetric and non-symmetric waveforms by considering both the rising and falling shape of the wave patterns. It separates the data into two groups that belong to the rising or falling edge of an oscillation period. These are then tested using a summation of Mann-Whitney tests for rising and falling slopes independently. Other commonly used algorithms in the field are compiled in an R package named MetaCycle [153], which merges JTK_CYCLE (JTK) [147], Lomb Scargle (LS) [144,154] and ARSER (ARS) [143]. LS periodogram is an extension of the Fourier-like Spectrum analysis with an additional extension for the detection of rhythmic pattern in an unevenly distributed time series. In a classical periodogram analysis, uniform sampling with Gaussian white noise allows the results to be normally distributed. However, for unequal sampling, the classical approaches for distinguishing periodic and non-periodic signals remains challenging. LS resolves this issue by estimation of frequency spectrum with a least-square approach based on sinusoidal fitting to the data points.

JTK (Jonckheere-Terpstra Kendall’s Tau) applies a ranking to gene expression values. It uses the Jonckheere-Terpstra’s trend test that aims at the detection of a repetitive change between different experimental groups based on a dependent variable such as mRNA expression values and integrates the time as an independent variable. The Kendall’s Tau method is used for the assessment of rank correlation for two independent quantities. Based on the pre-defined period length, JTK identifies an ideal combination of phases and periods to characterize the cycling patterns [147]. Empirical JTK, which is an extension of JTK, incorporates also non-symmetrical waveforms [146]. 

ARSER uses an autoregressive spectral domain approach, which detects and outputs oscillation parameters (period, phase and amplitude). It considers signal to noise (S/N) levels and uses harmonic regression to model the changes over time. In recent years, deep neural networks (DNNs) for the detection of circadian rhythmicity have been used in several studies [148,155]. BIO_CYCLE, which is also based on DNNs, works firstly by training an algorithm with periodic data. This data is then used to estimate period, amplitude, phase and phase lag of a new subset of training data [148]. It is suggested that BIO_CYCLE can outperform some of the other commonly used methods like JTK_CYCLE for the detection of circadian rhythmicity. However, as the method can only be applied via an interactive portal, its current application is limited due to the lack of freedom to optimize algorithm parameters for different dataset requirements.

Each of the above-mentioned methods has different pros and cons, which should be carefully evaluated before the application. One of the relevant aspects for the accurate assessment of circadian rhythms relies on how the algorithms handle noisy data, a problem commonly encountered in biological data. JTK_CYCLE performs well against low signal to noise (S/N) ratio and can be favored when there are outliers in the data set. It is also widely used as a comparison for the assessment of prediction accuracy in machine learning studies. ARSER is less affected by the level of noise and curve type bias, due to the combination of time-domain and frequency-domain analyses that underlie the algorithm but cannot deal with uneven sampling or with missing values. RAIN, which uses a ranking approach to the real gene expression values, works well for the detection circadian of rhythmic genes and considers also non-symmetrical waveforms. However, it does not work as robust as parametric approaches (such as harmonic regression) in terms of the identification of phases. LS, RAIN, e-JTK and BIO_CYCLE can work on unevenly sampled time-series data and can be favored in data sets with irregular sampling intervals.

A single method cannot be easily favored as the rhythmicity detection depends also on the filters applied to the data set while defining the rhythmic genes using *p-* and *q*-value thresholds for a particular rhythmicity method. For the comparison of different computational algorithms used in rhythmicity analysis, next we plotted the number of rhythmic genes detected in the above described time course data (E-MTAB-7779, [29], Figure 3D). We compared the intersection of the number of genes detected by these different algorithms, harmonic regression, RAIN, LS, JTK and ARS (*p* < 0.05, for all methods). Although LS was tested in the same data set, there were no significant oscillations detected, the same had been previously observed in another time-course data set using LS [139]. Our results showed that RAIN and harmonic regression shared the highest number of genes detected to be circadian expressed (Figure 3D) followed by JTK_CYCLE and ARS. These results highlight the relevance of the algorithm used for defining the set of rhythmically expressed genes. While for example the harmonic regression method is suitable for the detection of rhythmicity using cosine/sine functions, RAIN, which allows for the incorporation of non-parametric waveforms, can be used for the detection of circadian oscillations when rhythmicity patterns cannot be modelled using trigonometric functions. It should be noted that in our example only the significance of the fit to a 24 h period of the oscillations was used for the detection of circadian rhythmic genes. However, considerations of additional circadian properties such as phase shifts and relative amplitudes might also be used for comparisons of oscillating patterns between different experimental conditions or for a more curated selection of circadian expressed genes.

### 3.2. Machine Learning in Prediction of Circadian Time 

**Figure 4:**
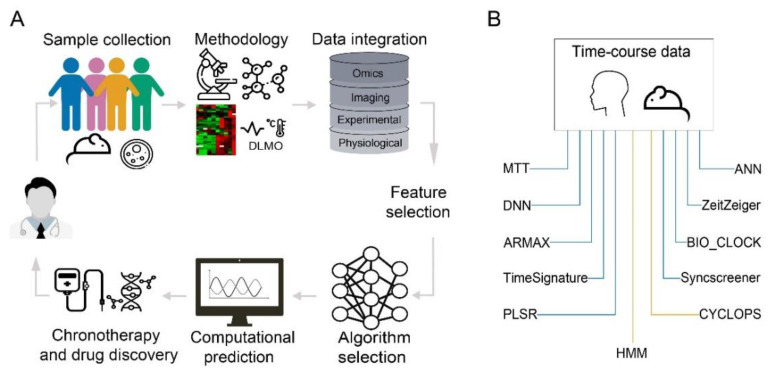
Schematic workflow of a computational prediction pipeline using machine learning based algorithms and possible applications in personalized medicine. (**A**) Inter-disciplinary data collected from human subjects, animal and cell line models are integrated using computational methods. The integrated data is then examined and features (variables) depicting potentially relevant information for the prediction model are selected. Based on the complexity of features and the prediction goal, an algorithm is selected and the computational prediction takes place. The prediction model can produce knowledge that serve as the basis for chronotherapy planning. (**B**) Schematic representation of supervised (blue lines) and unsupervised (yellow lines) machine learning algorithms used to predict circadian parameters based on genomics or physiological data.

Recent improvements in high-throughput screening and computational efficiency contributed to the application of machine learning in various areas of medical research [156,157,158,159] and drug discovery [160,161]. The amount of time-course based experiments on animal models has increased considerably and data sets are available at open source databases (e.g., EUCLOCK, CircadiOmics or GEO), which set the basis for the development of predictive time-dependent computational models (Figure 4A). However, available data sets are still limited for human biological samples and would be necessary for the further development of applications of circadian research for clinical usage. With the current methodologies, including the development of non-invasive sampling methods (as discussed in Section 2.1), we are likely to gain access to more time-course based data in the future especially from human subjects, for the development of more accurate predictive models. Nevertheless, machine learning holds already an enormous potential in the prediction of circadian parameters for subsequent usage in chronotherapy [162]. Machine learning based prediction methods are majorly categorized as supervised and unsupervised learning algorithms [163]. Supervised machine learning involves methods in which the model is trained on a specific set of input parameters or features, that are associated with a known outcome like the interpretation of Electrocardiograph (ECG) images and its association with risk assessment of a disease through individual characteristics (e.g., age, body mass index) [164]. On the other hand, unsupervised machine learning is not associated with a known outcome. In unsupervised learning, the model is trained on the unlabeled data, this means that only input data is provided for which no expected output is known. Unsupervised learning algorithms then find the underlying connections between datasets using approaches like clustering. In medicine, the application of unsupervised learning holds a great potential in finding common patterns (e.g., similar disease clusters) from patient’s electronic health records, where the specific output is not provided and thus can be used as a decision support tool for the diagnosis by the physician [165]. To explore the current status of machine learning in circadian research, we have included both genomics- and phenotype/physiological-based studies that have developed and/or utilized different algorithms for predictions applied to circadian research (Figure 4B).

#### 3.2.1. Application of Machine Learning Methodologies on Gene Expression Data

In the past years, different machine learning based algorithms were applied on high-throughput RNA-sequencing and microarray data obtained from different types of in vitro and in vivo mammalian model systems to predict various circadian variables [60,61,62,63,148,155,166,167,168,169,170]. Molecular-timetable (MTT) was the first reported supervised learning based method which predicted circadian time through the evaluation of a set of “time-indicating genes” from mouse liver gene expression data [166]. MTT trains a predictor by selecting features that have high periodicity (i.e., high correlation with a cosine curve of a 24 h period at any phase angle) and overall high variability. A set of time-indicating genes was defined based on the criteria of significant circadian rhythmicity and high amplitude. The peak time of the best-fitted cosine curve to the genome-wide expression profiles (obtained from DNA microarrays) of the time-indicating genes, was used as an estimation of the circadian time. The time-indicating genes included a set of more than 100 genes out of which some genes were *NR1D1*, *NR1D2*, *BMAL1* and *PER2*. The authors evaluated the accuracy of the MTT algorithm by measuring the absolute difference between the experimental sampling time and the estimated circadian time. The accuracy of MTT directly correlates with the number of identified rhythmic genes from genome datasets (>100 genes). Later, the MTT method was adapted to construct molecular timetables of blood metabolites using metabolomics data of mice plasma [171] and human plasma samples [172]. “TimeTeller” based on supervised learning was later developed to differentiate between functional and dysfunctional clock from mammalian microarray data [170]. The TimeTeller panel consisted of 10 clock genes (*BMAL1*, *NPAS2*, *PER1/2/3*, *NR1D1*, *NR1D2*, *CHRONO*, Thyrotrophic Embryonic Factor (*TEF*) and D Site-Binding Protein (*DBP*)). The combined expression of 10 clock genes was then used by the algorithm to calculate the likelihood curve, which depicted the single phase from the expression of 10 genes. The clock dysfunctional metric was then calculated as the ratio of the predicted phase by TimeTeller to the original time point of sample collection. The lower and higher clock dysfunctional metric represented a functional and dysfunctional clock, respectively. Other machine learning based algorithms like “BIO_CLOCK” were developed to predict the time at which the experiment took place [148]. The training for the BIO_CLOCK algorithm was carried out using previously established deep neural network methodologies [173,174]. This algorithm was developed using synthetic data from the BioCycle_Synth_ database and mouse gene-expression data from the CircadiOmics database. BIO_CLOCK can predict the time (within 1 h range) at which a particular sample from a transcriptomic based experiment was obtained. BIO_CLOCK works best when trained and tested on the data generated from the same tissue type. The authors reported a testing error when the trained model was derived using data from one tissue and then applied to a different tissue type for predictions. Considering the tissue-specific variation in circadian rhythm, the ZeitZeiger algorithm, which is based on supervised machine learning was developed using a microarray data set derived from 21 different mice organs [168]. ZeitZeiger was reported as a multi-organ predictor of internal time (circadian time). The phase predicted by ZeitZeiger was termed as the circadian time. The accuracy of ZeitZeiger was evaluated by analyzing the difference between the predicted circadian time and the real circadian time. Because of no true values of real circadian time in the datasets exist, the real circadian time was the time according to external cues (i.e., zeitgebers). The circadian time for each sample was standardized to be in 0–24 h range. CT0 corresponded to the sunrise in ZeitZeiger. The precision of ZeitZeiger (≤1 h) was compared against MTT (~3 h) and showed higher performance. ZeitZeiger was successfully tested on RNA-seq data of human blood samples for the prediction of circadian time and its accuracy was compared against DLMO phase values [60,62].

Often, publicly available human gene expression data sets contain missing values or lack of information on the sample collection time. Cyclic Ordering by Periodic Structure (CYCLOPS) algorithm was built to predict rhythmic patterns from high-dimensional human data containing missing information [169]. CYCLOPS utilizes evolutionary conservation and unsupervised machine learning to predict the phase of each sample. The authors validated CYCLOPS by testing the algorithm on genomic data from mouse liver and human cancer cell lines where the time of sampling was known and CYCLOPS predicted the phase of samples with an error of ≤2 h against the original time point. Intriguingly, the proof-of-concept of CYCLOPS was also demonstrated by authors in finding the best time for drug administration in an animal model.

To address the heterogeneity of data originating from different animal models and human studies, the partial least square regression (PLSR) method was developed. The authors have shown that this method predicted the DLMO phase with an error of ≤2 h when compared to the actual DLMO phase and the results obtained with the MTT algorithm [63]. The PLSR method can also work with only two samples for optimization. The diversity of gene expression platforms presents an obstacle to the applicability of a generalized prediction model. “TimeSignature,” a supervised learning method, was developed as a generalized predictor of circadian time from microarray and RNA-seq mammalian data and to overcome differences in clinical and experimental data [61]. TimeSignature was tested on four different transcription profiling studies of human blood. The authors demonstrated that the trained model created from one study can be applied without re-normalization to different studies. Moreover, cross-platform validation and accuracy of TimeSignature (≤2 h) outperformed ZeitZeiger (3 h) and PLSR (~2.25 h) in the prediction of circadian time when compared to the corresponding time of sampling.

#### 3.2.2. Application of Machine Learning on Non-Invasive and Phenotypic Measurements

Besides genomic data, many studies rely on indirect measurements like ambulatory recordings of wrist actigraphy and DLMO to predict circadian time and evaluate misalignments [175,176]. Several studies applied indirect and non-invasive measurements to develop a predictive model of phase [123,124,125,126,127,128,177]. Autoregressive moving average with exogenous inputs (ARMAX) model was developed using a supervised learning approach and predicted the DLMO phase using 24 h data obtained from ambulatory recordings [127]. Such recordings included wrist actigraphy, light exposure, electrocardiograms (ECG) and proximal/distal skin temperature data from healthy subjects. Additionally, saliva samples were collected to measure the actual DLMO phase as a reference. The model was trained using all possible combinations of features like heart rate variability, physical activity and light exposure. The difference between predicted DLMO phase and actual DLMO phase was defined as the prediction error. The best performing model was obtained using the combination of heart rate variability and light exposure data, which showed a prediction error of 39 min. The authors have later also tested ARMAX by incorporating additional data like skin temperature from the upper leg of healthy patients measured during two weeks and showed similar accuracy against the DLMO method [123]. Ambulatory measurements of body temperature, ECG, respiration and body movement from healthy subjects (number of subjects = 25) were also used to predict the phase using an artificial neural network (ANN) based model [128]. The measurements were taken maintaining the real-life conditions and without stressing the subjects. The ANN method outputted results with a prediction error of 41 min when compared to the predicted melatonin onset using the standard DLMO procedure. The ANN based model was later tested on shift workers (number of subjects = 28) for its generalizability [126]. Indeed, the model could predict the phase with a precision of ±2 h, compared to DLMO measurements, for 89% of individuals on diurnal schedules but showed poor performance when applied to datasets from individuals in night shift schedules.

Actigraphy is another non-invasive method to assess human sleep/wake cycles. A hidden Markov model (HMM) from unsupervised learning was applied to predict sleep/wake time in multi-ethnic individuals (number of subjects = 43) data [125]. The data used for the HMM algorithm were taken from the Multi-Ethnic cohort Study of Atherosclerosis (MESA; number of subjects = 2200) [178,179]. The HMM model showed higher accuracy (85.7% of 43 subjects) in prediction of sleep/wake cycles when compared against an Actiwatch device software (84.7% of 43 subjects). Indirect calorimetry (IC) measurements can also serve to gain insights into circadian rhythms. This method provided detailed information on the energy metabolism of mice, it evaluated energy expenditure through indirect calorimetry by measuring oxygen consumption with an open flow respirometric system [177]. The International Mouse Phenotyping Consortium (IMPC) provides indirect calorimetric data generated from different knock-out mouse models. “Syncscreener” was developed to predict phase using available calorimetric measurements from wild type and genetically altered mice. The algorithm could predict five genes associated with circadian misalignment among 750 mutant lines [177].

The gained information regarding predictions of the circadian phase, from established machine learning algorithms using genomics or physiological data, may be enhanced by the possible combination of both genomic and physiological data into one prediction model increasing the prediction power and driving the applicability of circadian information into the clinics.

### 3.3. Modelling Genetic Networks Related to Cancer and Circadian Time 

While a machine-learning algorithm typically constructs a relation between subject’s data and the personalized clock phenotype without detailed considerations of the underlying biological process, biological knowledge on the drug target-specific molecular network can add information and thus improve the predictive power of the model towards its usability in treatment timing. In this section, we review promising mathematical models of circadian biological processes, such as the transcriptional-translational networks underlying circadian rhythmicity as well as cancer pharmacokinetics and pharmacodynamics, that is, the dynamical action of pharmacological interventions on a molecular level, involving cell metabolism, cell cycle and toxicity. These types of mathematical models are built using ordinary differential equations (ODEs) and can be used as a pre-processing step for the machine-learning methodologies (reviewed in Section 3.2) or can be suitably extended to directly predict optimal treatment timing. In the following, we will focus on transcriptional-translational network mathematical models based on the mammalian SCN clock, given its pacemaker role and thus relevance for the determination of the circadian time.

Models of other mammalian tissues, such as liver [180], might be particularly interesting for tumor specific modelling of circadian rhythms, given their role in the regulation of drug metabolism and detoxification pathways. Various models also exist for non-mammalian organisms, see for example [181,182,183].

**Figure 5:**
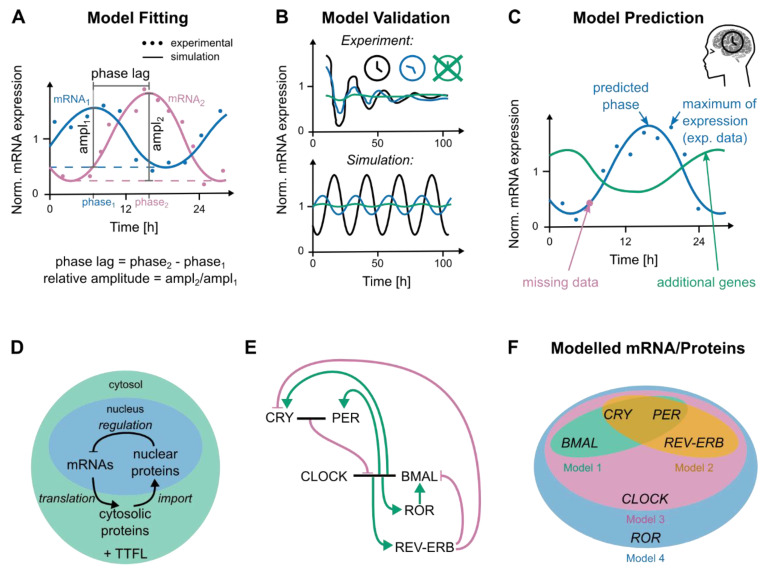
Basic mechanisms of genetic core-clock models. (**A**) Adapting the parameters of the model, the simulated model (blue and pink lines) can be fitted to experimental or clinical data (blue and pink dots). (**B**) After fitting, the model is validated by predicting, for example, whether or not oscillations are observed for known knock-out scenarios. (**C**) The model can be used to directly predict the circadian phenotype or to derive features for a subsequent prediction, such as additional data points or unobserved data. (**D**) The negative feedback loop in the circadian clock is based on an mRNA, which, translated to a cytosolic protein, is imported to the nucleus, where the nuclear protein inhibits the subsequent transcription of its target mRNA. (**E**) The basic interactions between different genes in the core-clock network. Each node represents a simplification of the translation/importing steps depicted in D. Green lines represent activation reactions, pink lines inhibitions and black lines complex formation. (**F**) Published molecular models of the core-clock implement different sets of core-clock genes. Models were selected for mammalian parameters and a focus on core-clock gene dynamics. Model 1, Becker-Weimann et al. 2004 [190]; Model 2, Forger et al. 2003 [189]; Model 3, Leloup et al. 2003 [188]; Model 4, Mirsky et al. 2009 [191]; Relógio et al. 2011 [23].

A model is a formal and often simplified or even incomplete, description of a biological process. The biological processes of interest for the prediction of circadian time for optimized cancer treatment is on one hand the mammalian core-clock (described in detail in Section 2) and on the other hand the processes related to the toxicity and efficacy of the particular cancer treatment. When simulating a mathematical model in the computer, the output, for example temporal profiles of RNA or protein expression, can be compared to experimentally measured molecular values, obtained from biological samples and used for parameter estimation and model fitting. Typical models of gene regulatory networks consist of differential equations. The resulting dynamic behavior, which simulates the expression profiles of genes and proteins, is specified by a set of parameters, such as transcription or degradation rates, chosen such that the model output fits the experimental results (Figure 5A). Once the model is fitted, deliberate perturbations in specific parameters can mimic experimental scenarios, such as knockouts of specific genes or blocking the binding of a specific protein to its target [8]. On the one hand, this allows for reproduction of experimental outcomes beyond those used for fitting, showing that the model captures a particular biological mechanism (Figure 5B). On the other hand, choosing a perturbation not yet carried out experimentally, results in a prediction of future experimental outcomes, which raises new hypotheses and drives the planning of new experiments. When these predictions are tested experimentally, the results can be used to recursively refine the model—a continuous interaction between experiment and model that enhance our biological understanding and can also reduce the need for animal research. Often data from cell lines that show a dampening of the oscillation as in Figure 5B is used to fit models. The dampening comes from a continuous dispersion of the cells that were initially synchronized (by external agents, for example, serum shock). Models can capture this behavior by explicitly modelling a noise source, which will lead to a similar dispersion in phase when simulating multiple outputs of the model.

To assess optimal timing of cancer treatment, models can in principle be used in two ways, one directly resulting in a prediction and the other indirectly, as a pre-processing step for subsequent predictions. For example, a core-clock model could (i) increase the temporal resolution of the measured time-series by extrapolating additional data points and thus help to derive peak time and oscillation amplitude, (ii) predict missing data, as a common precondition for machine learning is complete data or (iii) it could derive the temporal dynamics of mRNA or proteins that were not measured experimentally but which are included in the model (Figure 5C–E). Such simulated elements (mRNA or proteins) with a high predictive power could, for example, be added to the set of experimentally measured genes to improve predictions. Models can also be used directly for predictions by additionally implementing a medically relevant output such as time-dependent toxicity for a particular drug. The implementation of the chosen output can be a simple linear step on top of the original model or can involve additional network elements. An example for a linear relation is the prediction of the best cancer treatment time in mice, where the expression profiles of two core-clock genes, *Rev-erbα* and *Bmal1*, were used to model the toxicity of the medication as a predictor for optimal timing [93]. Additional network elements can for example result from joining two models. For example, extending a core-clock model with a model for pharmacokinetics and pharmacodynamics provides additional information on the causal chain linking expression data to toxicity. In this section, we review available models of the core-clock and treatment-associated transcriptional-translational networks.

The mammalian clock has been modelled on different levels of detail. An example of a model is with few details is the response of the clock network to zeitgebers such as light (Figure 2). Exposure to light shifts the phase of circadian rhythms. The length of the shift depends on the timing of the light exposure. The relation between timing of light and induced shift, the so-called phase response curve, allows for a prediction of the overall change in the circadian phase by a summation of the individual changes due to light exposure [122,184]. Compared to predictions based on gene expression [61,63], predictions with an error of approximately one hour are astonishingly precise given the simple underlying model [122], potentially because people intuitively adapt their daily routine and thus also their light exposure (and activity) to their circadian phenotype. For bedridden patients entrained to clinical routine, whose internal circadian rhythm are reduced in amplitude [185], predictions will likely be worse. For these patients, the prediction of circadian time based on gene expression data has been proposed [60]. We here suggest that predictions might be improved by taking advantage of additional biological information. For example, an almost anti-phase relationship is observed for the mRNA of *BMAL1* and *PER2*, as sketched for the traces of mRNA_1_ and mRNA_2_ in Figure 2F [186]. If a patient shows instead an in-phase relationship, a problem with at least one of these genes seems likely. A crucial question remains: How can we test in general whether patient data agree with the healthy state established by experimental results? As the core-clock model incorporates this knowledge, subjects can be considered as having a functional core-clock if their circadian profile can be fitted by the core-clock model. On the other side, fitted parameters with unexpected values might hint at which part of the clock is malfunctioning, for example, an import rate close to zero could hint at a dysfunctional import process. The role of the unrealistic parameters might even help to identify which pathways are disturbed.

Regulatory gene network-based models or molecular models for the circadian clock, date back to work by Goodwin in 1965 [187], who conceived a three-variables oscillator model involving a negative feedback loop (Figure 5D), for a review see [183]. The negative feedback results from the inhibition of mRNA transcription by its own protein product, resulting in repeated waxing and waning of the model variables. Current clock models consider a set of interacting core-clock genes [23,188,189,190,191,192] (Figure 5E,F). The mRNA is translated into a cytosolic protein, which, once imported to the nucleus, acts as a transcription factor closing the loop (Figure 5D). The time needed to reach a certain concentration of the cytoplasmic protein, its subsequent nuclear import and binding to the target gene to initiate transcription introduces delays to the system, which eventually add up to the 24 h-period observed for circadian oscillations.

Detailed molecular models of the core-clock are based on interaction networks similar to the example represented in Figure 5E, which is mostly based on the model from Relógio et al. [23]. As often done, the network in Figure 5E merges members of a gene family into a single variable (e.g., PER instead of PER1, PER2 and PER3). To translate such a network into a mathematical model, a few established methods are available (reviewed in [8,193]). Most approaches make use of ordinary differential equations to describe the molecular interactions, that is, equations which relate an element’s growth or decay with its causes. For example, the amount of nuclear protein increases with the import of cytosolic protein and decreases, for example, due to degradation, export and complex formation. While the step from the gene network to the equations is straightforward, the thereby established model has unknown parameters, whose identification is a prerequisite for the application of the model. Early clock models were fitted to show realistic periods and the ability for entrainment [188,189], recent core-clock models base the parameter estimation on (increasing) sets of experimental observations [23,191,192]. For finding appropriate parameters of core-clock models, two approaches have been used. Some authors retrieve experimental values, via a curated literature search, to identify as many parameters as possible and manually fit only the remaining parameters based on experimental data and educated guesses [23,190]. Other models use automated approaches, which repetitively improve on the parameters to fit the model on available experimental data [191,192]. The latter approach is considered as more objective, however, there might be more than a single parameter combination that leads to the experimental behavior or phenotype. Due to the nature of complex systems, the algorithm might find an optimum of *in silico* parameters that involves biologically unrealistic values. Some fitting procedures allow to specify biological constraints, such as used by Ballesta et al. [194] or Kim et al. [192]. Unrealistic parameter values are also prevented by manual curation of the parameters. Here, the difficulty is that the information is collected from different sources and might thus not fit together. Personalized core-clock models use the parameter sets of published mammalian models as an initial guess and try to reduce the range of admissible parameters based on experimental data. A relatively large range of parameters in the models leads to qualitatively similar behavior, as the oscillation inherits salient properties from a limited number of oscillation mechanism [23,192,195,196]. Noteworthy is an approach which does not provide a single set of parameters but considers a whole ensemble of model fits simultaneously [197].

The step from genes to treatment has been attempted for one cancer medication, irinotecan (CPT11), whose activity is controlled by core-clock-regulated genes [93,198,199]. Irinotecan administration induces a cellular pathway which eventually leads to the formation of a cell-toxic complex involving TOP1 (topoisomerase 1). This enzyme catalyzes the transient breaking and re-joining of a single strand of DNA altering the topologic states of DNA during transcription and is extremely important during DNA repair [200,201]. TOP1, as well as further elements of the pharmacokinetic pathway (ABC-transporters, CES2 (Carboxylesterase 2), UGT1A (UDP Glucuronosyltransferase Family 1 Member A Complex Locus)) are regulated by the core-clock, thus the toxicity of irinotecan for synchronized cells, which are interpreted as healthy, can be minimized by appropriately timed administration, while for unsynchronized cancer cells the timing is irrelevant [198,199]. The model furthermore allows to identify the most critical processes involved in cytotoxicity [199]. While a general prediction of optimal irinotecan timing can be based on cell lines [198,199], the prediction could potentially be improved by personalized transcription-translational networks. A personalized cancer-specific model of the core-clock should for example include the mutations in core-clock genes seen in around 4% of cancer patients [44]. Besides the irinotecan network, the addition of a cell-cycle network could be interesting for treatment by timing medication to the cell cycle of non-cancer cells, which likely have a different clock [25]. Given the range of molecular time-dependent processes that the circadian clock regulates, including metabolism, DNA repair and the cell cycle, it has the potential to act as a tumor suppressor [25,46]. Indeed, several clock-controlled genes show a strong association with cancer and cancer progression, in particular genes related to metabolism [26,28,44]. In reverse, cancer also impacts the core-clock network [48], which, when implemented into a combined model, will result in a complex interaction between the core-clock and the cancer network. An even more complete model would in addition model the micro-environment of the tumor cells, for example fibroblasts and their impact on tumor cells [36].

## 4. Discussion, Limitations and Conclusions 

Several therapeutic interventions are likely to be time-of-day-dependent and their efficacy could potentially be optimized by individual tailoring, based on the internal rhythms of the patient. However, multiple bottlenecks need to be overcome, in order to advance chronotherapeutic applications. As of 2016, less than 0.2% of all clinical trials registered under https://clinicaltrials.gov include chronobiological aspects [202]. The individual differences of the ideal dosing times, partially attributed to variations in gender and age groups, create a challenge for the translation of circadian research findings into clinical practice. Another key barrier in preventing the widespread adoption of time-of-day-dependent therapeutic approaches in clinics is the lack of a standard method for circadian phase/internal time determination, which is preferably non-tedious, user-friendly and affordable. Therefore, for the successful application of the knowledge of the internal clock in chronotherapy and chrono-medicine, the method should have (a) high accuracy, sensitivity and reliability regarding individual assessment of circadian parameters in the clinic and (b) it should be user friendly and non-invasive, so that it could be performed at home by the patients themselves or in a routine examination at the hospital [203]. One possible solution to overcome this limitation is the usage of wearable devices, which can gather physiological information at high-temporal resolution and from different sources to monitor and predict circadian variations in patients (see Section 2.1).

The study of the core-clock, clock-controlled genes and their related biological and molecular processes, in human subjects and animal models, has strongly contributed to gaining insights into the circadian dysregulation underlying severe diseases including cancer [204,205,206,207,208,209,210]. Determining the circadian time and the circadian profile of a healthy subject and detecting possible abnormalities or malfunctions of the biological clock which may trigger disease or increase its severity remains a challenging task, in particular when asking for a mechanistic understanding on which clinical trials may be based. A wealth of knowledge may be expected from future high-throughput screenings interpreted with bioinformatic expertise. Recent studies advanced our ability to predict circadian parameters and individualized circadian time, from genomics and physiological data using machine learning algorithms. Several algorithms like ZeitZeiger and TimeSignature could predict circadian variables from inter-species data sets obtained from high-throughput sequencing. On the other hand, algorithms like ARMAX and ANN could predict circadian phase from physiological data. 

At the current state of the art, circadian phase is predicted with an error of 1 to 2 h. To gauge the quality of prediction, this value has to be compared with the error that would result from using the external time directly as prediction for the circadian time. As the reported range of circadian time is typically less than 6 h, as shown in previous studies (e.g., [61]), it seems reasonable to assume that circadian phases of humans are distributed in a Gaussian bell-shaped curve with a standard deviation of 1.5–2 h, such that 95% of tested individuals have a circadian phase within an interval of 6 h [162]. Drawing circadian phases from this distribution and assigning as “prediction” the mean of the distribution, results in a standard deviation of 1.5–2 h for the error, which is potentially on the same order of magnitude as some prediction errors reported in the literature. This shows that the estimation of circadian time still has potential to be improved. It is likely that the combination of genomics and physiological data may result in the development of more accurate machine learning models for circadian time prediction. Such models can also be used for the stratification of subjects (e.g., for clinical trials) based on their circadian profiles, as well as for the optimization of individualized therapies. Further improvement might result from a combination of machine learning with modelling. Testing circadian parameters determined by machine learning methodologies on a mathematical model might also help to validate predictions for their clinical application. The estimated circadian time can be used to predict optimal treatment timing. Given the current low precision of circadian phase predictions, it might be more useful to replace circadian phase by a direct prediction of optimal treatment timing. This might have the advantage of reduced error accumulation compared to the detour via circadian time and could facilitate the usage of ultradian rhythms, which are lost when focusing on circadian time [211,212]. For direct predictions of optimal treatment timing, mathematical models might be particularly useful, for example they can help in optimizing the time for drug delivery with low toxicity outcome. 

## 5. Future Perspectives 

The endogenous circadian clock regulates numerous processes in our cells. The circadian machinery targets thousands of genes and leads to the oscillatory expression of more than 40% of genes across all different mammalian tissues. At least 50% of these genes are drug targets of FDA (Food and Drug Administration) approved drugs. The potential impact of timing in drug development and treatment cannot be ignored. Yet more research is needed to bring such knowledge into the clinics. Clinical studies with larger cohorts of subjects, stratified according to their circadian profiles, are still lacking. The experimental methods used for sampling biological material and their analysis must be improved to allow for the robust assessment of the biological clock and the subsequent generation of clinically relevant predictions. Another challenge in the field is the lack of one common methodology for the detection of rhythmic patterns. Our analysis using different algorithms for the detection of circadian oscillations in time course data revealed distinct numbers of rhythmically expressed genes across methods, this may result in irreproducibility of the observed results, which should be addressed in the future. The usage of machine learning algorithms and predictive mathematical models of regulatory networks in circadian research enables to utilize and extrapolate biological data and to identify predictive circadian parameters. Such knowledge may lead to the identification of biomarkers within circadian expressed genes and can ultimately be used for accurate estimates of drug administration timing. The difference between core-clock and peripheral clock poses an open challenge for cancer therapy. While current predictions focus on the central clock, the peripheral clock of the cancerous tissue might be more important for its treatment. As tumor removal is hopefully done at a single time point, genomics-based predictions, which only require a single time point are particularly promising to assess the relevant tissue-specific internal time. Most studies use machine learning to predict circadian time, based on a relatively small training set of a few subjects, with the drawback of low statistical power [1]. Machine learning becomes more powerful with increasing data sets. The establishment of a large data set which could be used to train prediction algorithms is best achievable by a joint effort, for example via the establishment of an international database fed with clinical time-course data on different patients. In this regard, a promising initiative is the Clinical Trials Information System (CTIS) of the European Medical Agency, once the clinical trial regulation comes into application. Time-course data could be collected as part of the normal clinical routine, if, for example, daily blood drawing was performed at different times of the day. But not only clinicians, also pharmaceutical companies are asked for their contribution by providing information on treatment timing. Testing treatment success at different treatment time points to increase in the patient’s well-being will hopefully be worth the increased financial costs resulting from such an extension of drug testing. The characterization and monitoring of our internal circadian clock will potentially lead us towards a tailor-made optimized treatment of cancer with less side effects and increased efficacy.

## Figures and Tables

**Table 1:** Most commonly used questionnaires utilized for chronotype estimation.

Questionnaire	Abbreviation	Number of Questions	Results
Morningness-Eveningness Questionnaire	MEQ	19	five categories: definite evening type, moderate evening type, neither type, moderate morning type and definite morning type
Composite Scale of Morningness	CSM	13	three categories: evening type, intermediate type and morning type.
Munich ChronoType Questionnaire	MCTQ	32	population-specific continuous distributions featuring different categories

**Table 2:** Cancer-related drugs from the top-100 best-selling drugs list that target circadian genes and have half-life < 6 h. Information extracted from [42].

Drug	Indications	Drug Target Genes	Organs in Which Targets Oscillate
Filgrastim	Acute myeloid leukemia	*Csf3r*	lung
Rituximab	Non-Hodgkin’s lymphoma	*Fcgr2b*, *Ms4a1*, *Fcgr3*	liver, kidney, skeletal muscle
Bevacizumab	Colorectal cancer, Non-small cell lung cancer	*Fcgr2b*, *Vegfa*, *Fcgr3*	heart, brown fat, liver, kidney, skeletal muscle, aorta
Trastuzumab	Breast cancer	*Fcgr2b*, *Erbb2*, *Egfr*, *Fcgr3*	heart, liver, kidney
Imatinib	Chronic myeloid leukemia	*Ptgs1*, *Kit*, *Slc22a2*, *Abcg2*, *Pdgfra*, *Pdgfrb*, *Ddr1*, *Abca3*, *Abl1*, *Ret*, *Abcb1a*	lung, heart, brainstem, white fat, adrenal gland, brown fat, liver, kidney
Pemetrexed	Non-small cell lung cancer	*Tyms*, *Atic*, *Gart*, *Slc29a1*	lung, heart, brainstem, brown fat, liver, kidney, aorta
Filgrastim	Acute myeloid leukemia	*Csf3r*	lung
Capecitabine	Breast cancer, colorectal cancer	*Cda*, *Tymp*, *Tyms*, *Ces1g*, *Dpyd*	lung, adrenal gland, liver, brown fat, kidney, aorta

**Table 3:** Summary of clinical trials conducted to examine the effect of time-of-day adjusted cancer treatment.

Type of Cancer	Type of Intervention:	Reference
Brain metastasis in non-small-cell lung carcinoma	Radiosurgery	[101,102]
Multiple brain metastases	Radiotherapy	[103]
Breast cancer	Radiotherapy	[104]
Breast carcinoma, hepatocellular carcinoma, Cholangiocarcinoma	Chemotherapy	[105]
Painful bone metastases	Radiotherapy	[106]
Cervical carcinoma	Radiotherapy	[107]
Colorectal	Chemotherapy	[5,6,98,108,109,110]
Head and neck	Radiotherapy	[111,112]
Oral squamous cell carcinoma	Chemotherapy	[113]
Prostate adenocarcinoma	Radiotherapy	[114]
Soft tissue, bone sarcoma	Chemotherapy	[115]
Nasopharyngeal carcinoma	Chemotherapy	[116,117,118]
Non-small-cell lung carcinoma	Chemotherapy	[119]
High-grade glioma	Chemotherapy	[120]
